# Dexamethasone as Abortive Treatment for Refractory Seizures or Status Epilepticus in the Inpatient Setting

**DOI:** 10.1177/2324709619848816

**Published:** 2019-05-18

**Authors:** Alexander B. Ramos, Roberto A. Cruz, Nicole R. Villemarette-Pittman, Piotr W. Olejniczak, Edward C. Mader

**Affiliations:** 1 Louisiana State University Health Sciences Center, New Orleans, LA, USA

**Keywords:** refractory seizures, status epilepticus, antiepileptic drugs, inflammation, anti-inflammatory, steroid, dexamethasone

## Abstract

Refractory seizures or status epilepticus (RS/SE) continues to be a challenge in the inpatient setting. Failure to abort a seizure with antiepileptic drugs (AEDs) may lead to intubation and treatment with general anesthesia exposing patients to complications, extending hospitalization, and increasing the cost of care. Studies have shown a key role of inflammatory mediators in seizure generation and termination. We describe 4 patients with RS/SE that was aborted when dexamethasone was added to conventional AEDs: a 61-year-old female with temporal lobe epilepsy who presented with delirium, nonconvulsive status epilepticus, and oculomyoclonic status; a 56-year-old female with history of traumatic left frontal lobe hemorrhage who developed right face and hand epilepsia partialis continua followed by refractory focal clonic seizures; a 51-year-old male with history of traumatic intracranial hemorrhage who exhibited left-sided epilepsia partialis continua; and a 75-year-old female with history of breast cancer who manifested nonconvulsive status epilepticus and refractory focal clonic seizures. All patients continued experiencing RS/SE despite first- and second-line therapy, and one patient continued to experience RS/SE despite third-line therapy. Failure to abort RS/SE with conventional therapy motivated us to administer intravenous dexamethasone. A 10-mg load was given (except in one patient) followed by 4.0- 5.2 mg q6h. All clinical and electrographic seizures stopped 3-4 days after starting dexamethasone. When dexamethasone was discontinued 1-3 days after seizures stopped, all patients remained seizure-free on 2-3 AEDs. The cessation of RS/SE when dexamethasone was added to conventional antiseizure therapy suggests that inflammatory processes are involved in the pathogenesis of RS/SE.

## Introduction

Status epilepticus (SE) is defined by the International League against Epilepsy (ILAE) as a condition resulting either from the failure of the mechanisms responsible for seizure termination or from the initiation of mechanisms, which (after time point t_1_) lead to abnormally prolonged seizures and which (after time point t_2_) result in long-term consequences including neuronal death, neuronal injury, and alteration of neuronal networks, depending on the duration and seizures type.^1,2^ A classification system for SE was recently proposed by the ILAE based on 4 criteria (axes): semiology (Table 1), etiology, age, and electroencephalography (EEG) correlates.^1^ The following information must be included in describing the EEG in SE: (1) spatial distribution: generalized or bisynchronous, lateralized, bilateral independent, multifocal); (2) name of pattern: periodic discharges, rhythmic delta activity, or spike-and-wave/sharp-and-wave plus subtypes: (3) morphology: sharpness, number of phases (eg, triphasic), absolute and relative amplitude, polarity; (4) temporal features: onset (sudden vs gradual), prevalence, frequency, duration, daily pattern, dynamics (evolving, fluctuating, or static); (5) modulation: stimulus-induced versus spontaneous; and (6) effects of interventions, such as antiepileptic drug (AED) administration.^1,3^

**Table 1.** Classification of Status Epilepticus^a^.

A. With Prominent Motor Symptoms	B. Without Prominent Motor Symptoms (ie, Nonconvulsive SE, NCSE)
A.1. Convulsive SE (CSE, synonym: tonic-clonic SE) A.1.a. Generalized convulsive A.1.b. Focal onset evolving into bilateral convulsive SE A.1.c. Unknown whether focal or generalized A.2. Myoclonic SE (prominent epileptic myoclonic jerks) A.2.a. With coma A.2.b. Without coma A.3. Focal motor A.3.a. Repeated focal motor seizures (Jacksonian) A.3.b. Epilepsia partialis continua (EPC) A.3.c. Adversive status A.3.d. Oculoclonic status A.3.e. Ictal paresis (ie, focal inhibitory SE) A.4. Tonic status A.5. Hyperkinetic SE	B.1. NCSE with coma (including so-called “subtle” SE) B.2. NCSE without coma B.2.a. Generalized B.2.a.a. Typical absence status B.2.a.b. Atypical absence status B.2.a.c. Myoclonic absence status B.2.b. Focal B.2.b.a. Without impairment of consciousness (aura continua, with autonomic, sensory, visual, olfactory, gustatory, emotional/psychic/experiential, or auditory symptoms) B.2.b.b. Aphasic status B.2.b.c. With impaired consciousness B.2.c. Unknown whether focal or generalized B.2.c.a. Autonomic SE

Abbreviations: SE, status epilepticus; NCSE, nonconvulsive SE; CSE, convulsive SE; EPC, epilepsia partialis continua.

a The table shows axis 1 of the classification of SE as proposed by the International League against Epilepsy Task Force on Classification of SE and published in 2015 (see: *Epilepsia*. 2015;56:1515-1523).^1^ Adapted with permission.

Convulsive SE (CSE; A.1.a. in Table 1) is characterized by prolonged (>5 minutes) convulsions, that is, episodes of excessive abnormal muscle contractions, usually bilateral, which may be sustained or interrupted.^1,4^ The best time point estimates for CSE are: t_1_ = 5 minutes, t_2_ = 30 minutes.^1^ Nonconvulsive SE (NCSE; SE.B category in Table 1) includes all SE subtypes without prominent motor manifestations (Table1). The best time point estimates for focal NCSE with impaired consciousness (B.2.b.c in Table 1) are: t_1_ = 10 minutes, t_2_ > 60 minutes.^1^ In this article, we will simply refer to the B.2.b.c subtype of NCSE as NCSE. Refractory status epilepticus (RSE) has no universally accepted definition. Some definitions specify a minimum number of AEDs to which SE fails to respond (eg, 2 or 3) or a minimum time over which SE persists despite adequate treatment (eg, 1 or 2 hours).^5^ A common definition is the following: SE persisting despite treatment with at least one benzodiazepine and at least one non-benzodiazepine AED.^6^ Each type of SE is fundamentally related to a specific type of short-duration seizure.^7^ While short-duration seizures do not meet the definition of SE, non-SE seizures that frequently occur despite adequate AED treatment may actually need the same treatment as their SE counterparts. We arbitrarily define refractory seizures (RS) as frequently recurring (>10 episodes over 24 hours) short-duration (<t_1_) seizures that cannot be controlled with at least one benzodiazepine and at least one non-benzodiazepine AED (same criteria for RSE).

RS or status epilepticus (RS/SE) is a major challenge in the inpatient setting for a number of reasons. First, there is no incontrovertible evidence in humans that all forms of sustained epileptiform activity in the electroencephalogram (EEG) will result in clinically significant brain injury and enduring functional impairment in addition to that which has been caused by the primary brain lesion, systemic disturbances, and medical interventions.^8-10^ Second, the physiological basis and pathological implications of various scalp EEG patterns remain controversial and it is not always easy to decide where some EEG patterns would lie in the ictal-interictal continuum.^11-13^ Third, it is not always possible to abort RS/SE with currently available AEDs even when several drugs are combined and administered at maximum (nonanesthetic) doses.^14,15^ Fourth, aggressive treatment of RS/SE with general anesthesia, intubation, and intensive care unit (ICU) monitoring exposes the patient to the risk of medical complications, increases the cost of health care, and puts a strain on limited hospital resources.^16,17^ In the United States, patients with RS/SE are often managed in the ICU where continuous EEG (CEEG) monitoring is performed.

SE, notably CSE, is an emergency that must be aborted immediately when encountered in the field, clinic, ambulance, emergency room (ER), or inpatient setting. CSE can be diagnosed clinically whereas NCSE requires EEG confirmation. Because SE can be difficult to abort, physicians who are faced with this challenge must be aware of a few caveats. While it is well known that inducing SE in laboratory animals can cause irreversible brain injury, the results of most animal studies cannot be extrapolated to human SE, particularly NCSE.^18-20^ Several clinical studies have correlated SE with increased probability of unfavorable functional outcome or death. However, the increase in morbidity or mortality rate can also be attributed to other factors, including the etiology of SE, the age of the patient, the patient’s overall state at presentation (coma and GCSE are predictors of poor outcome), and the length of ICU treatment.^21-23^ There is also no solid evidence that rapid termination of NCSE can affect prognosis independent of the effects of etiology and other factors mentioned above.^24,25^ One might argue that these uncertainties justify aggressive treatment of RS/SE in the ICU, but intubation, anesthesia, and other ICU interventions may be related to an even higher risk of complications and mortality than SE itself, especially NCSE.^16,17,26,27^

Abortive treatment of RS/SE in the inpatient setting can be divided into 3 treatment lines: first-line treatment employs fast-acting benzodiazepines, second-line treatment involves the administration of intravenous (IV) AEDs, and third-line treatment is carried out in the ICU with general anesthetics.^28^ Only the first-line treatment of CSE is currently supported by a high level of evidence.^29^ The optimal treatment for RS/SE and the sequence of administration of AEDs and anesthetic agents, especially in patients with NCSE, remains controversial.^30^ Despite the first 2 treatment lines, about a third of SE patients continue seizing and progress to refractory SE and about half of patients with refractory SE progress to super-refractory SE.^31^ This motivated experts to try drugs with no SE indication (eg, other AEDs or anesthetics, magnesium, corticosteroids, and sex hormones) and other measures, such as hypothermia, electroconvulsive therapy, transcranial magnetic stimulation, and resective surgery.^32^ Of these therapeutic options, adding an anti-inflammatory steroid appears to be the most practical and most promising approach for now. In the last decade, nearly 2 dozen applications of anti-inflammatory steroids (dexamethasone or methylprednisolone) in our Epilepsy Center resulted in RS/SE abortion after failure of conventional inpatient RS/SE treatment protocols. Our recent (within the last 24 months) successful abortive experiences with 4 adult RS/SE patients who received IV dexamethasone are presented here.

## Case Series

All 4 patients were seen in consultation by our adult neurology inpatient service and all had RS/SE that was controlled with IV dexamethasone after failure of first- and second-line treatment of SE. Institutional review board review of a project summary resulted in an exemption status from institutional review board approval and continued monitoring. Demographic and clinical information are summarized in Table 2. Detailed clinical information and timelines are presented in the text for each patient. SE types, seizure types, and EEG findings are described based on the ILAE and ACNS recommended nomenclatures.^1-3^

**Table 2.** Demographic and Clinical Summary of Patients Presented^a^.

Patient Series #, Age (in Years), and Gender	Preexisting Disorder of the Brain and Relevant PAST Medical Conditions	Refractory Seizures or Status Subtypes^b^ and Response to Conventional Therapy (A, Aborted; P, Persisted)	Conventional Hospital Therapy to Abort Seizures	Intravenous Dexamethasone Dosing Schedule	Time of Seizure Abortion With Dexamethasone
1, 61/female	Temporal lobe epilepsy with bilateral mesial temporal sclerosis	Nonconvulsive status epilepticus w/o coma w/ impaired consciousness (P)	Lorazepam, levetiracetam, lacosamide valproate	10 mg loading dose	3 days after the first dose of dexamethasone
		Oculoclonic status (A)		5.2 mg q6h (3 days)	
				5.2 mg q6h (2 days)	
2, 56/female	H/o traumatic brain injury: left frontal parenchymal and subdural hemorrhage	Epilepsia partialis continua (A)	Lorazepam, levetiracetam, lacosamide	10 mg loading dose	3 days after the first dose of dexamethasone
		Refractory focal clonic seizures (P)		4 mg q6h (3 days)	
3, 51/male	H/o traumatic brain injury: right frontal and parietal hemorrhage	Epilepsia partialis continua (P)	Lorazepam, levetiracetam, lacosamide	No loading dose	3 days after the first dose of dexamethasone
				4 mg q6h (3 days)	
4, 75/female	H/o breast cancer: no MRI evidence of structural brain lesion	Nonconvulsive status epilepticus w/o coma w/ impaired consciousness (P)	Propofol, levetiracetam, fosphenytoin, midazolam, lacosamide	10 mg loading dose	4 days after the first dose of dexamethasone
		Refractory focal clonic seizures (P)		4 mg q6h (6 days)	

a We arbitrarily defined refractory seizures as frequently-recurring (>10 episodes over 24 hours) short-duration (<t_1_) seizures that cannot be controlled with at least one benzodiazepine and at least one non-benzodiazepine antiepileptic drug. Seizures that were immediately aborted are not included in the table.

b See Report of the ILAE Task Force on Classification of Status Epilepticus (*Epilepsia*, 2015)^1^ and ILAE 2017 operational classification of seizure types (*Epilepsia*, 2017)^2^.

**Figure 1.**
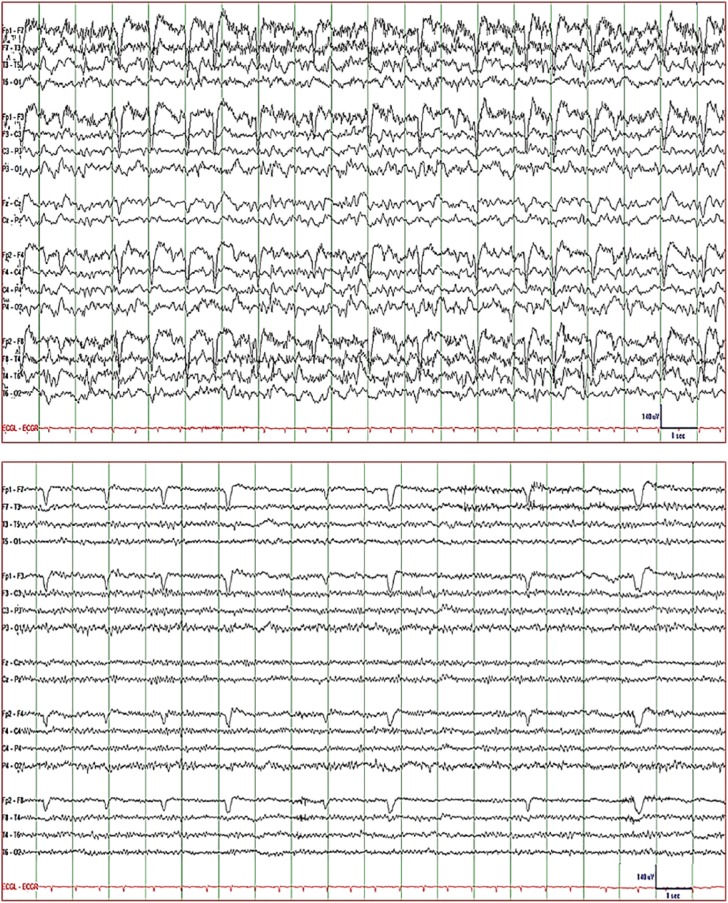
EEG of Patient 1. *Top*: generalized 2 to 3 Hz semirhythmic delta activity superimposed on rhythmic theta and alpha activity with patient delirious and perseverating consistent with NCSE and with patient blinking every second on average (see eye blink artifacts) consistent with oculoclonic status. *Bottom*: electrographic (and clinical as seen on video) resolution of status epilepticus 3 days after dexamethasone was added to conventional inpatient antiepileptic therapy. *Display parameters*: longitudinal bipolar montage (from top to bottom: left-mid-right-ECG), digital filter bandpass of 1 to 70 Hz, and 60-Hz notch filter turned on; voltage-time scale is included in the tracing.

Patient 1 is a 61-year-old female with mesial temporal lobe epilepsy and bilateral hippocampal sclerosis who presented with a 3-day history of delirium. She arrived in the ER mildly confused with constant eye blinking. EEG revealed generalized 2 to 3 Hz semirhythmic delta activity superimposed on rhythmic theta and alpha activity with eye blink artifacts occurring at a rate of ~1/s (Figure 1: top). The findings were consistent with NCSE without coma with impaired consciousness (SE:B2bc; Table 1) and with oculoclonic status (SE:A3d; Table 1). After treatment with lorazepam 4-mg IV and levetiracetam 2000-mg IV, she was admitted to the ICU and CEEG was started. Oculoclonic status ceased but NCSE persisted on levetiracetam 1500-mg IV q12. Lacosamide 300-mg IV was loaded the next day followed by 200-mg IV q12. On day 3, valproate 2000-mg IV was also loaded followed by 1000-mg IV q12h, but this was stopped 2 days later because of hyperammonemia. On day 4, the dose of levetiracetam was increased to 2000-mg IV q12h. She developed third-degree atrioventricular block and symptomatic bradycardia, which resolved when the dose of lacosamide was reduced to 100-mg IV q12h. Despite all these measures, NCSE persisted but third-line treatment with anesthesia was not justified since she remained awake and conversant (albeit confused). Instead, dexamethasone was started on day 5. A 10-mg IV load was administered followed by 5.2-mg IV q6h. Three days after starting dexamethasone, CEEG showed complete resolution of epileptiform activity (Figure 1: bottom); and her mental status also started to normalize. Dexamethasone was continued for 2 more days at a lower dose of 5.2-mg IV q12h before it was finally discontinued. She remained on levetiracetam and lacosamide with no seizure recurrence.

**Figure 2.**
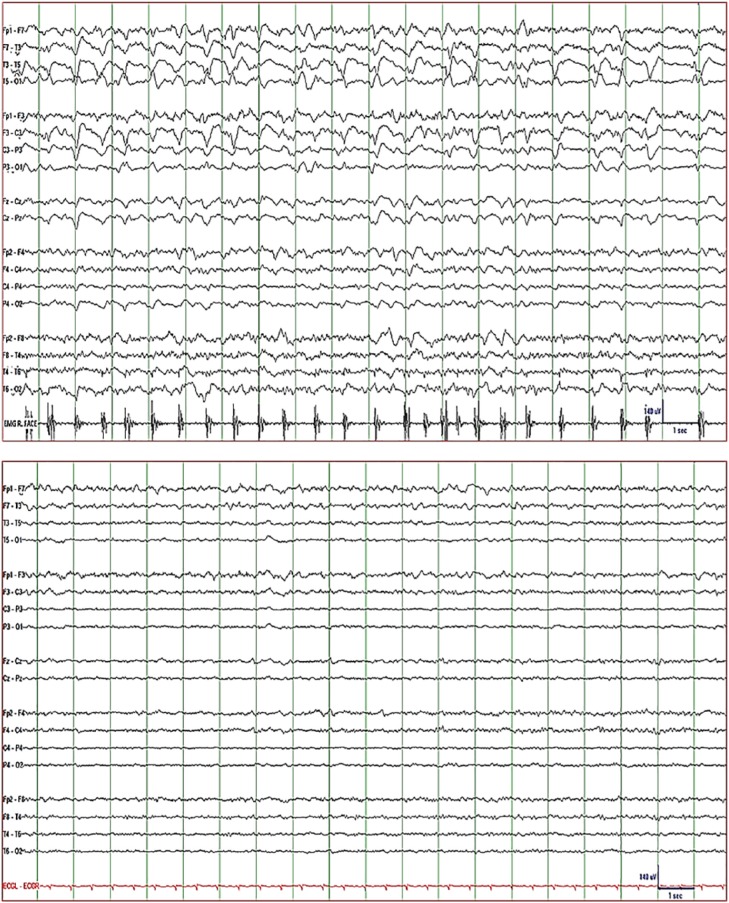
EEG of Patient 2. *Top*: lateralized periodic discharges (sharp delta waves) over the left hemisphere time-locked to the myoclonic jerks of the right hand and face (surface EMG recorded over right face) consistent with epilepsia partialis continua. Note fluctuation of discharge rate from 1.5 to 2/s to 0.5 to 1/s. *Bottom*: electrographic resolution of periodic discharges 3 days after dexamethasone was added to conventional antiepileptic therapy coinciding with complete control of clinical seizures. *Display parameters*: longitudinal bipolar montage (from top to bottom: left-mid-right-EMG/ECG), digital filter bandpass of 1 to 70 Hz, and 60-Hz notch filter turned on; voltage-time scale is included in the tracing.

Patient 2 is a 56-year-old female with a recent history of seizures attributed to left frontal hemorrhage from head trauma 2 months prior to admission. She was taking levetiracetam 1000-mg PO q12h. She was found unresponsive and brought to the ER where she had a 60-second episode of focal to bilateral tonic-clonic seizure. Initial treatment consisted of lorazepam 4-mg IV and levetiracetam 1500-mg IV loading dose followed by 500-mg IV q12h. EEG on day 2 showed left frontal interictal sharp waves. Her mental status improved and she became less somnolent. She was seizure-free for 4 days before she had a 30-second focal clonic seizure involving the right face and hand with impaired awareness. She became inattentive and somnolent again so levetiracetam was increased to 1000-mg IV q12h. On day 5, she started having continuous right face and hand jerking and EEG showed 0.5 to 1/s lateralized periodic discharges (sharp waves) over the left hemisphere that were time-locked to the jerks (Figure 2: top). The findings were consistent with epilepsia partialis continua (EPC; SE:A3b; Table 1). Brain MRI revealed acute left temporoparietal infarction in addition to old traumatic brain lesions. After lacosamide 100-mg IV q12h was added, myoclonic jerks stopped and she became more alert. On day 9, she started having focal aware clonic seizures, which resembled the initial EPC except for lack of persistence (t_1_ < 30 seconds). The dose of lacosamide was increased to 150-mg IV q12h, but focal clonic seizures continued to occur frequently (~1/hour). Dexamethasone 10-mg IV was loaded followed by 4-mg IV q6h. After 3 days on dexamethasone, she became seizure-free (Figure 2: bottom). Dexamethasone was discontinued 25 hours after the last seizure. She remained seizure-free on levetiracetam and lacosamide.

**Figure 3.**
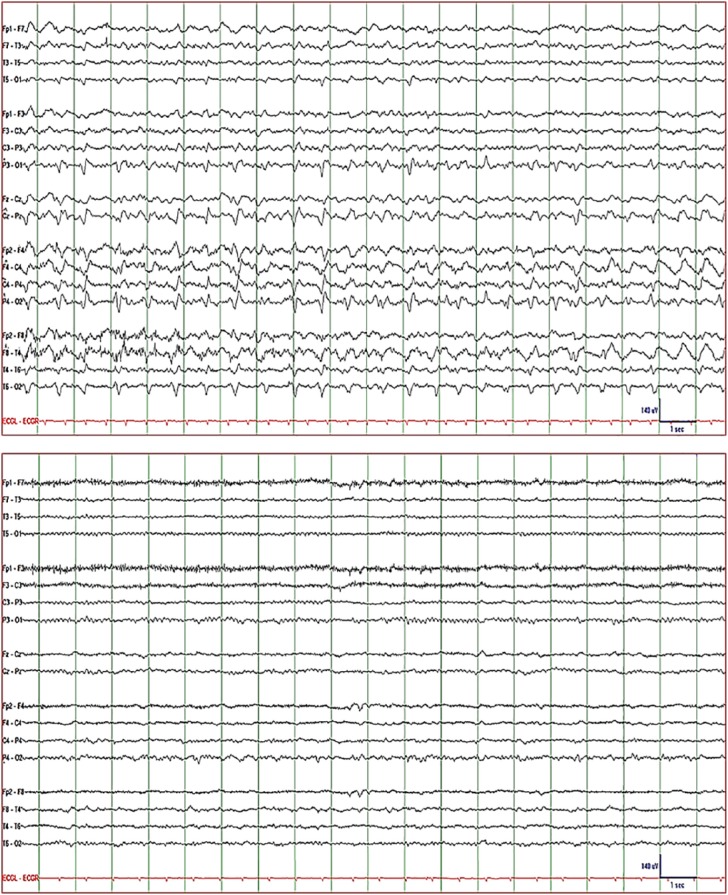
EEG of Patient 3. *Top*: 0.5 to 1/s lateralized periodic discharges (sharp/delta waves) with maximum voltage over the right frontocentral superimposed on irregular slow waves and time-locked to the myoclonic jerks of the left face, arm, and leg consistent with epilepsia partialis continua. *Bottom*: electrographic (and clinical as seen on video) resolution of epilepsia partialis continua 3 days after dexamethasone was added to conventional antiepileptic regimen. *Display parameters*: longitudinal bipolar montage (from top to bottom: left-mid-right-ECG), digital filter bandpass of 1 to70 Hz, and 60-Hz notch filter turned on; voltage-time scale is included in the tracing.

Patient 3 is a 51-year-old male with a history of right frontal and parietal hemorrhage due to head trauma 6 months prior to admission. He had seizures in the past and was recently admitted due to tonic-clonic seizures, which was controlled with levetiracetam, lacosamide, and carbamazepine. He was discharged on these 3 AEDs, but only took levetiracetam 1000-mg PO q12h prior at home. He started exhibiting left face, arm, and leg jerking at home. On admission, EEG showed 0.5 to 1/s periodic sharp and delta waves superimposed on irregular slow waves over the right hemisphere with maximum voltage over the right frontocentral region (Figure 3: top). The discharges were time-locked to the left face, arm, and leg jerks consistent with EPC (SE:A3b; Table 1). To abort EPC, the dose of levetiracetam was increased to 2000-mg IV q12h and lacosamide was started at a dose of 200-mg IV q12h. Left face and arm jerking stopped, but EPC persisted with jerking restricted to the left leg. Dexamethasone was started at 4-mg IV q6h (loading dose was not given). Seizures stopped completely 3 days after initiating dexamethasone (Figure 3: bottom). Dexamethasone was discontinued the next day and she remained seizure-free on levetiracetam and lacosamide.

**Figure 4.**
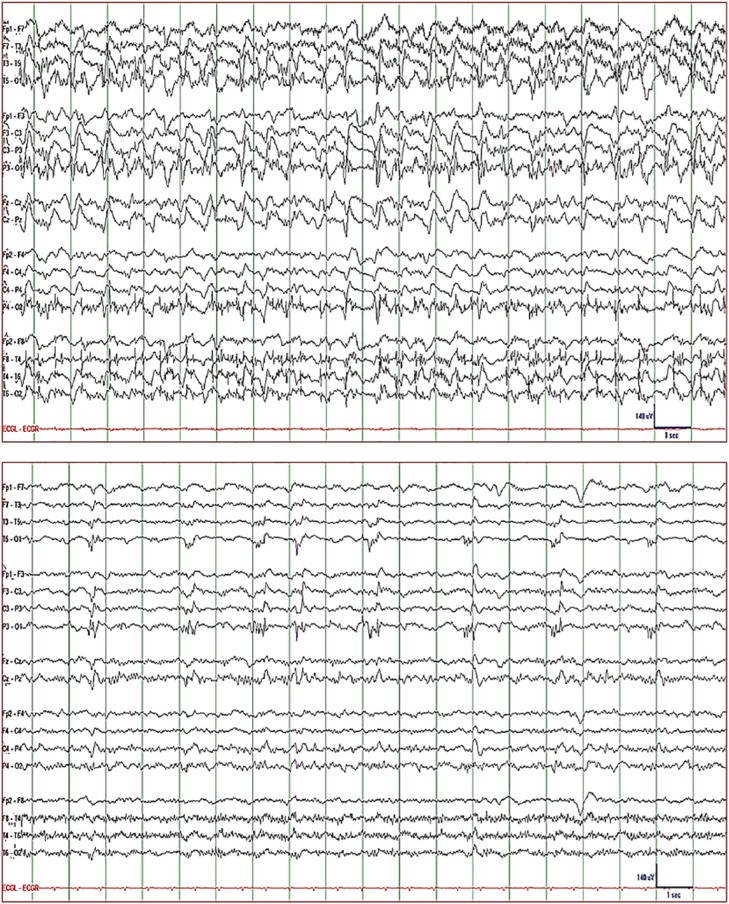
EEG of Patient 4. *Top*: 1 to 2/s lateralized (left > right) periodic discharges with sharp morphology and superimposed on semirhythmic delta activity recorded while the patient was stuporous with intermittent episodes of right lower extremity jerking findings that are consistent with NCSE without coma with impaired consciousness and focal clonic seizure. *Bottom*: Persistence of left frontocentral 0.3 to 0.5/s periodic discharges approximately 5 days after dexamethasone was added and the patient was already seizure-free. *Display parameters*: longitudinal bipolar montage (from top to bottom: left-mid-right-ECG), digital filter bandpass of 1 to 70 Hz, and 60-Hz notch filter turned on; voltage-time scale is included in the tracing.

Patient 4 is a 75-year-old female with a history of metastatic breast cancer who presented in stupor with intermittent 60-second episodes of right lower extremity jerking. EEG showed 0.5 to 1/s lateralized (left > right) periodic discharges with sharp morphology and superimposed semirhythmic delta activity (Figure 4: top). The findings were consistent with NCSE without coma with impaired consciousness (SE:B2bc; Table 1) with recurrent focal clonic seizures (t1 30-60 seconds). She was intubated for airway protection and propofol was started at 10-µg/kg/min IV. She was also loaded with 1500-mg IV of levetiracetam followed by 1000-mg IV q12h. Brain MRI was normal. In the ICU, she continued to have focal clonic seizures (1-2/hour) and CEEG showed persistent NCSE. Fosphenytoin 2000-mg IV was loaded followed by 150-mg IV q8h. Propofol was uptitrated but she became hypotensive at 40 µg/kg/min. Midazolam drip was started and burst suppression was sustained for 2 days with 60 to 80 mg/kg/min of IV midazolam. Every time midazolam was weaned off, epileptiform discharges reappeared. Lacosamide 750-mg IV q12h IV was added. CEEG showed persistent NCSE with periodic sharp waves appearing more localized over the left frontocentral region. Focal clonic seizures also started to involve the right face and arm in addition to the leg. On day 6, dexamethasone 10-mg IV was loaded followed by 4-mg IV q6h. Four days after dexamethasone was started, all clinical seizures stopped but 0.3 to 0.5/s lateralized periodic discharges persisted in EEG (Figure 4: bottom). Dexamethasone was continued for 2 more days after she stopped seizing. She remained seizure-free on levetiracetam, lacosamide, and phenytoin.

## Discussion

Seizure control can be achieved by eliminating all factors that promote neuronal hyperexcitability and hypersynchrony. In practice, this is not always feasible in the acutely ill patient. In the inpatient setting, the general approach is to abort seizures immediately with rapidly acting AEDs, followed by maintenance therapy to prevent seizure recurrence. Unfortunately, RS/SE—seizures that persist despite conventional treatment with maximal doses of multiple AEDs—is not uncommon in the inpatient setting. Our case series shows that adding IV dexamethasone to conventional antiseizure therapy (lorazepam, levetiracetam, lacosamide, valproate, fosphenytoin, and/or anesthetic agents) can help abort RS/SE. Clinical and electrographic seizures stopped in all 4 patients 3 to 4 days after dexamethasone was started, and all patients remained seizure-free on 2 or 3 AEDs after dexamethasone was discontinued 1 to 3 days after all seizures stopped.

Inflammation plays an important role in seizure disorders. This role can be viewed from the perspective of autoimmunity, epilepsy, and pharmacoresistant seizures. The link between autoimmunity and seizure disorders is obvious in the case of autoimmune encephalitis where autoantibodies target biomolecules involved in neuronal signaling.^33^ However, even if autoimmunity is not the primary mechanism causing the seizure disorder, inflammation and immune processes may still be important in the pathogenesis of epilepsy.^34,35^ There is mounting evidence that inflammatory mediators released from the brain and peripheral immune cells may be involved in generating seizures (ictogenesis) and emergence of epileptic networks (epileptogenesis).^36,37^ Finally, it is known that some inflammatory mediators play a role, not only in generating but also in maintaining seizures, implying that these inflammatory mediators contribute to the pharmacoresistance of RS/SE.

Animal research has shown that anti-inflammatory therapy given during or shortly after SE reduces the severity of the ensuing epilepsy, as reflected by a reduction in the incidence, frequency, severity, and spread of seizures and by a decrease in brain cell loss and comorbidities.^32^ Nonetheless, we will emphasize animal experiment results indicating that brain inflammation occurs during SE and plays a role in driving seizures. Inflammatory mediators can lower the seizure threshold in animals by acting on neuronal receptors altering membrane excitability or by acting on genes inducing the transcription of proteins involved in synaptic plasticity.^38,39^ In rodents, various inflammatory response molecules or signaling pathways, such as the interleukins IL-1β and IL-1R1, toll-like receptor-4 (TLR4), cyclooxygenase-2 (COX-2) and prostaglandins, and complement, contribute to the onset and recurrence of stimulus-provoked seizures.^40-42^ Manipulation of some of these inflammatory pathways may reduce the incidence and recurrence of SE.^32^ For example, IL-1 receptor antagonists reduced the incidence, delayed the onset, and shortened the duration of pilocarpine-induced SE.^43^ Anakinra, a specific IL-1 receptor antagonist used to treat rheumatoid arthritis, caused a transient decrease in spike frequency when injected 3 hours after electrically induced SE.^44^ Antagonists of the P2X7 receptor in immune and other cells reduced the duration of acute SE.^45^ In an animal model of pharmacoresistant SE, co-administration of P2X7 receptor antagonists and benzodiazepines suppressed SE, presumably by reducing microglia activation and IL-1β levels in the forebrain.^46^ Injecting dexamethasone prior to pilocarpine in rats reduced the number of rats developing SE and, in rats that developed SE, the onset of SE was delayed and mortality was prevented; blood-brain barrier damage was also significantly reduced by dexamethasone.^47^ These results support our choice of dexamethasone (Decadron) over methylprednisolone (Solu-Medrol). In reality, we tend to choose dexamethasone for patients with no underlying autoimmunity since this drug is familiar to most physicians involved in the care of patients with RS/SE. There are, however, differences between dexamethasone, methylprednisolone, and other corticosteroids that physicians might have to consider when using anti-inflammatory steroids as adjunctive therapy for seizures.^48^

Human RS/SE is most likely influenced by inflammatory mediators, but few studies directly address this issue. Extensive extravasation of albumin has been detected in patients who died during SE indicating disruption of the blood-brain barrier.^49^ Signs of pronounced focal inflammation, including strong IL-1β expression, intense gliosis, and minimal lymphocytic infiltration, were found in the temporal cortex resected from a patient with refractory SE.^50^ The endogenous control of brain inflammation may be inadequate during SE.^32^ Children with febrile SE have elevated serum levels of IL-1β and IL-6; and children with febrile SE have higher HMGB1 levels as well compared with controls with fever but no seizures.^51^ The cerebrospinal fluid levels of the cytokines IL-6, IL-8, and CXCL10 were much higher in patients with refractory SE due to febrile infection-related epilepsy syndromes than in patients with other inflammatory brain disorders.^52^ The fact that adrenocorticotropic hormone (ACTH) and prednisolone are effective in West syndrome imply that inflammatory mechanisms are important in the pathophysiology of infantile spasm.^53^

Epilepsy pharmacotherapy relies heavily on seizure prevention with AEDs, but this approach should not be exclusive. An understanding of the mechanisms in which inflammatory mediators promote the formation of epileptic circuitry can lead to novel therapies for suppressing epileptogenesis directly.^54-56^ A related issue in need of urgent attention is the current approach to RS/SE in the inpatient setting. Immunotherapies, such as corticosteroids, plasma exchange, intravenous immunoglobulins, rituximab, and cyclophosphamide, are, by and large, reserved for seizure disorders with underlying autoimmune mechanisms.^57,58^ The use of immunomodulators to control RS/SE in the typical seizure patient with no autoimmune disorder deserves investigation. Controlled studies must be conducted to determine whether broad-spectrum anti-inflammatory steroids, such as dexamethasone or methylprednisolone, can help abort RS/SE and obviate intubation and anesthesia. The case series presented here only provides anecdotal evidence for stimulating discussion and perhaps further experimental investigations. These patients were treated based on their presenting and persistent situations, not in the systematic way required for scientific evidence.

## Conclusion

Despite an increase in the availability of fast-acting AEDs and anesthetic agents, aborting RS/SE in the inpatient setting can still be challenging. Multiple AEDs administered at maximal doses can fail to abort RS/SE. Because standard AED regimens may fail to abort RS/SE, and because intubation and anesthesia are not without risks, clinicians must have other options to treat RS/SE. Our case series shows that anti-inflammatory agents, in particular dexamethasone, may satisfy this need. Furthermore, our case series suggests a key role for inflammatory or immune factors in generating and perpetuating seizures, or at least drug-resistant seizures, in humans.
